# Assessments of Health Utilities in Patients With Metabolic Dysfunction-Associated Steatohepatitis: Cross-Walk Between Disease-Specific Chronic Liver Disease Questionnaire, Short Form SF-6D, and EuroQol EQ-5D Instruments

**DOI:** 10.1016/j.gastha.2025.100642

**Published:** 2025-02-07

**Authors:** Zobair M. Younossi, Maria Stepanova, Yestle Kim, Stephen Dodge, Dominic Labriola, Rebecca Taub, Fatema Nader

**Affiliations:** 1 The Global MASH Council, Washington, District of Columbia; 2 Beatty Liver and Obesity Research Program, Inova Health System, Falls Church, Virginia; 3 Madrigal Pharmaceuticals, Conshohocken, Pennsylvania; 4 Center for Outcomes Research in Liver Disease, Washington, District of Columbia

**Keywords:** Health-Related Quality of Life, Disease Burden, QALY, Cost-Utility Assessment, MASLD

## Abstract

**Background and Aims:**

The EuroQol-5D (EQ-5D) is a commonly used measure of health utilities to calculate quality-adjusted life years. For the clinical trials that use Chronic Liver Disease Questionnaire-nonalcoholic fatty liver disease (CLDQ-NAFLD) or Short Form-36 (SF-36), ability to convert the health-related quality of life scores (CLDQ-NAFLD or SF-36) to EQ-5D scores provides a valuable method to estimate health utility.

**Methods:**

Baseline data of noncirrhotic metabolic dysfunction-associated steatohepatitis (MASH) patients were used in this study. We used 2 cross-walk algorithms to estimate EQ-5D scores. The first algorithm used 6 domains of CLDQ-NAFLD in a fractional logistic model to yield EQ-5D estimates. The other algorithm included calculation of SF-6D utility scores from SF-36 items, which were fed into a regression model that estimated EQ-5D scores from SF-6D scores.

**Results:**

There were 883 MASH patients with CLDQ-NAFLD and SF-36 data: 25% ≥65 years, 44% male, 80% obese (body mass index >30), 67% type 2 diabetes, 62% F3 fibrosis, and 38% F1B/F2 fibrosis. The mean estimated EQ-5D scores were 0.851 (standard deviation = 0.146) according to CLDQ-NAFLD-based algorithm and 0.853 (standard deviation = 0.097) according to the SF-36-based algorithm. The correlations between the 2 estimated EQ-5D scores were up to +0.74. Similar to the total sample, the differences between the mean EQ-5D estimates using either calculation method did not exceed 0.012 in all studied subgroups (by age, sex, obesity, type 2 diabetes, and fibrosis stage).

**Conclusion:**

Both cross-walk algorithms for the calculation of the EQ-5D utility scores in MASH patients were estimable with CLDQ-NAFLD or SF-36 instruments. A high positive correlation was seen between the total score and subgroup estimates using either method.

## Introduction

Metabolic dysfunction-associated steatohepatitis (MASH) is the most common cause of liver disease leading to adverse clinical outcomes including cirrhosis, hepatocellular carcinoma, and liver-related mortality.^1^^,^^2^ In addition to its clinical burden, MASH causes significant economic burden as well as impairment of health-related quality of life (HRQL) and other patient-reported outcomes (PROs).^3^^,^^4^ In 2024, a novel thyroid beta receptor agonist (resmetirom) was approved for treatment of noncirrhotic MASH with moderate to advanced liver fibrosis (F2 and F3), in conjunction with lifestyle interventions.^5^ In a phase 3 clinical trial (MAESTRO-NASH), resmetirom not only met the dual primary histologic endpoints but was also associated with improvement of some aspects of PROs as measured by the generic Short Form-36 (SF-36) and disease-specific CLDQ-NASH.^6^

Health utilities in patients with chronic diseases are becoming a standard part of the generic PRO assessment.^7^, ^8^, ^9^, ^10^, ^11^, ^12^ By quantifying health outcomes in terms of utilities, health-care providers and policymakers can perform cost-utility analyses to determine the value of different treatments and interventions and to quality-adjust outcomes (eg, calculate quality-adjusted life years) for economic and disease burden studies.^13^^,^^14^ From its definition, health utilities are supposed to reflect patients' preference for a health status.^15^ Health utilities can be measured directly through techniques such as standard gamble or time trade-off which are, however, impractical in clinical studies.^16^^,^^17^ Given that, a number of indirect methods for estimating health utilities have been developed, including those based on specifically designed or preexisting PRO instruments.^18^^,^^19^ One method to estimate health utilities is via EuroQol-5D (EQ-5D) scores,^20^ which have been widely used in different populations and settings and were recommended for quantifying health utilities by the National Institute for Health and Care Excellence in the United Kingdom and the Institute for Clinical and Economic Review in the United States.^21^, ^22^, ^23^

While using the specifically designed EuroQol EQ-5D-5L instrument is the only way to directly measure the EQ-5D utility scores, it is sometimes not available or not feasible in a particular study due to the overall questionnaire burden or missing for post hoc analysis of historic PRO data. Given that EQ-5D is a generic PRO measure that should correlate with other aspects of HRQL and/or other PROs, it may be possible to approximate the EQ-5D scores from other PRO instruments. In fact, the calculation of health utility scores from the SF-36 generic questionnaire (the SF-6D scores) is one example of how preference-based utility scores can be calculated from PRO instruments.^24^ Similarly, having methods for estimating health utility scores from other PRO instruments may be useful for covering gaps in knowledge about disease burden and the value of interventions, especially in the context of highly prevalent chronic diseases with various treatment options.

Since EuroQoL-5D-5L was not administered in the MAESTRO-NASH Phase 3 trial, the aim of this study was to use baseline PRO data from the trial to approximate EQ-5D scores via other PRO instruments in patients with noncirrhotic MASH.

## Methods

### Study Population

For this post hoc study, baseline PRO data collected from noncirrhotic MASH patients who were enrolled in MAESTRO-NASH multicenter Phase 3 clinical trial (NCT03900429) were used. To be enrolled, the patients were required to have ≥3 metabolic risk factors, histologic evidence of MASH, and fibrosis stage F1B or F2 or F3; the full lists of inclusion/exclusion criteria have been previously published.^5^ Enrollment in the trial had been performed before the change in nomenclature from NASH to MASH was finalized, so all included subjects were additionally assessed for meeting the criteria for MASH.

### Cross-Walk Mapping Algorithms for Estimating EQ-5D Utility Scores

In the parent study, the PROs were assessed using the Chronic Liver Disease Questionnaire-nonalcoholic fatty liver disease (CLDQ-NAFLD; 36 items, 6 domains) and Liver Disease Quality of Life instruments; the latter included SF-36 as its first 36 items.^25^^,^^26^ At the parent study baseline, the instruments were completed by the patients on the first day of treatment with resmetirom prior to the initiation of any treatment-related activities. Only patients who completed at least one of the 2 PRO instruments at baseline were included in this study (the study sample). Using those, we applied 2 different mapping algorithms to estimate baseline EQ-5D scores in the study sample.

The first previously published algorithm used 6 domains of CLDQ-NAFLD in a fractional logistic model to yield EQ-5D estimates.^27^ In that study, a fractional model (a model with possible outcomes bounded between 0 and 1) with several knots (points of joining piecewise regression models, allows for flexible modeling) was chosen based on its accuracy in cross-validation.^27^ Its subtype that was also evaluated in this study used the total CLDQ-NAFLD score (rather than the 6 domain scores) in a similarly designed logistic model to estimate EQ-5D utility scores.^27^

For the second algorithm, we used a historic sample of MASH patients from our Global MASH registry and databases for whom baseline SF-36 and EQ-5D data were available.^28^ Enrollment to the registry had been performed before the change in nomenclature from NASH to MASH was finalized, so all included subjects were additionally assessed for meeting the criteria for MASH. For that sample, we calculated SF-6D utility scores from the items of SF-36 using a previously published nonparametric Bayesian method.^29^ The SF-6D scores were further fed into a generalized regression model with a polynomial link function that would fit the EQ-5D scores from the SF-6D scores. The resulting fitted model was then applied to the SF-6D scores (similarly derived from the SF-36 items) of the study patients to yield the EQ-5D estimates.

### Statistical Analysis

Clinico-demographic parameters of the study sample were summarized as n (%) or mean ± standard deviation (SD). The 3 EQ-5D estimates (returned by the modes that used the CLDQ-NAFLD total scores, the CLDQ-NAFLD domain scores, or the SF-6D scores, respectively) were evaluated for correlation with each other and then in predefined clinical subgroups (by age, sex, the presence of obesity, type 2 diabetes [T2D], and fibrosis stage). The scatter plot and the Bland-Altman plot were created for qualitative assessment of the correlations. Independent association of the estimated EQ-5D scores returned by the models with patients’ demographic and clinical parameters was assessed by generalized regression models.

All analyses were conducted in SAS 9.4 (SAS Institute, Cary, NC). All participants provided written informed consent before enrollment in the parent study. This post hoc study was done in accordance with the ethical principles of the Declaration of Helsinki and was consistent with the International Conference on Harmonisation Good Clinical Practice and applicable regulatory requirements. The institutional review boards or independent ethics committees of each study center approved the study and all amendments.

## Results

There were 883 MASH patients with CLDQ-NAFLD and SF-36 data who were included in this study: 25% were aged ≥65 years, 44% male, 72% non-Hispanic White, 80% obese (body mass index [BMI] >30), 67% had T2D, 62% had F3 fibrosis, and 38% had F1B or F2 fibrosis (Table 1).

**Table 1:** Demographic and Clinical Parameters of the Study Sample (Noncirrhotic MASH With Fibrosis)

	Male	Female	*P*	All
N	391 (44.3%)	492 (55.7%)		883
Age, y	55.9 ± 11.3	57.1 ± 10.7	.08	56.6 ± 11.0
Age ≥65 y	93 (23.8%)	126 (25.6%)	.53	219 (24.8%)
Non-Hispanic White	291 (75.6%)	334 (68.4%)	.0202	625 (71.6%)
Non-Hispanic Black	6 (1.6%)	11 (2.3%)	.46	17 (1.9%)
Hispanic	67 (17.4%)	122 (25.0%)	.0068	189 (21.6%)
Asian	11 (2.9%)	15 (3.1%)	.85	26 (3.0%)
Other race/ethnicity	10 (2.6%)	6 (1.2%)	.13	16 (1.8%)
Enrolled in the U.S.	242 (61.9%)	345 (70.1%)	.0101	587 (66.5%)
BMI, kg/m2	35.7 ± 6.3	35.8 ± 7.1	.80	35.7 ± 6.8
Obese (BMI >30)	321 (82.1%)	387 (78.7%)	.20	708 (80.2%)
Type 2 diabetes	253 (64.7%)	342 (69.5%)	.13	595 (67.4%)
Hypertension	315 (80.6%)	377 (76.6%)	.16	692 (78.4%)
Hyperlipidemia	294 (91.6%)	340 (87.9%)	.11	634 (89.5%)
Fibrosis stage F1B	23 (5.9%)	22 (4.5%)	.34	45 (5.1%)
Fibrosis stage F2	123 (31.5%)	169 (34.3%)	.36	292 (33.1%)
Fibrosis stage F3	245 (62.7%)	301 (61.2%)	.65	546 (61.8%)
HbA1C, %	6.56 ± 1.12	6.58 ± 1.03	.53	6.57 ± 1.07
Total cholesterol, mg/dL	173.6 ± 44.4	183.1 ± 47.2	.0033	178.9 ± 46.2
HDL, mg/dL	39.0 ± 12.2	47.2 ± 12.0	<.0001	43.6 ± 12.8
LDL, mg/dL	102.4 ± 37.1	107.8 ± 39.0	.06	105.4 ± 38.3
Triglycerides, mg/dL	208.7 ± 159.3	175.7 ± 112.2	.0002	190.3 ± 136.0
ASCVD score	19.7 ± 12.5	11.7 ± 10.3	<.0001	15.1 ± 11.9
ALT, U/L	57.0 ± 32.3	53.1 ± 33.1	.0025	54.8 ± 32.8
AST, U/L	38.3 ± 19.6	42.5 ± 26.2	.10	40.6 ± 23.6
ALP, U/L	66.3 ± 20.6	79.5 ± 26.4	<.0001	73.6 ± 24.9
CAP, dB/m	356.6 ± 35.2	339.8 ± 37.6	<.0001	347.3 ± 37.5
MRI-PDFF, %	17.9 ± 6.7	17.5 ± 6.8	.30	17.7 ± 6.8
FIB-4 score	1.44 ± 0.70	1.42 ± 0.71	.41	1.43 ± 0.71
ELF score	9.73 ± 0.90	9.78 ± 0.86	.33	9.76 ± 0.88
Liver stiffness by TE, kPa	13.3 ± 6.3	13.3 ± 6.9	.81	13.3 ± 6.6
CLDQ-NAFLD: Abdominal symptoms	5.83 ± 1.28	4.98 ± 1.60	<.0001	5.36 ± 1.52
CLDQ-NAFLD: Activity/energy	5.73 ± 1.25	5.13 ± 1.39	<.0001	5.40 ± 1.36
CLDQ-NAFLD: Emotional health	5.61 ± 1.18	5.24 ± 1.25	<.0001	5.41 ± 1.23
CLDQ-NAFLD: Fatigue	4.99 ± 1.35	4.45 ± 1.47	<.0001	4.69 ± 1.44
CLDQ-NAFLD: Systemic symptoms	5.40 ± 1.17	4.88 ± 1.27	<.0001	5.12 ± 1.25
CLDQ-NAFLD: Worry	5.88 ± 1.26	5.40 ± 1.47	<.0001	5.61 ± 1.40
Total CLDQ-NAFLD	5.57 ± 1.03	5.01 ± 1.15	<.0001	5.26 ± 1.13
SF-6D utility	0.720 ± 0.135	0.661 ± 0.125	<.0001	0.687 ± 0.132

ALP, alkaline phosphatase; ALT, alanine aminotransferase; ASCVD, atherosclerotic cardiovascular disease; AST, aspartate aminotransferase; CAP, controlled attenuation parameter; CLDQ-NAFLD, Chronic Liver Disease Questionnaire-nonalcoholic fatty liver disease; ELF, enhanced liver fibrosis; FIB-4, fibrosis-4; HbA1c, hemoglobin A1c; HDL, high-density lipoprotein; LDL, low-density lipoprotein; MRI-PDFF, magnetic resonance imaging proton density fat fraction; TE, transient elastography.

After applying the previously published CLDQ-NAFLD-based models^27^ to this study sample, we found that the mean estimated EQ-5D scores in the total sample were 0.848 (SD = 0.135) according to the total score-based model and 0.851 (SD = 0.146) according to the domain scores-based model. Since the 2 CLDQ-NAFLD-based models returned similar EQ-5D estimates, we further refer to the domain scores-based model for comparison to the SF-6D-based model described below.

The historic sample of patients with MASH from the Global MASH registries who had both EQ-5D and SF-6D values (N = 1623) (Table A1)^28^ was used to train a regression model that would predict EQ-5D from SF-6D. The resulting regression model was fitted as follows: EQ-5D estimate = −0.16054 + 2.29536∗SF-6D −1.15066∗(SF-6D∗∗2). The root mean squared error of the fitted model was 0.1015, and the correlations of the observed EQ-5D values with the observed SF-6D and fitted EQ-5D were +0.68 and +0.71, respectively. When applied to the study sample, the mean estimated EQ-5D score returned by this model was 0.853 (SD 0.097).

The correlations between the estimated EQ-5D scores returned by the CLDQ-NAFLD domain scores-based model and the SF-6D-based model were +0.704 (Pearson’s) and +0.739 (Spearman’s), respectively (Figure). Similar to the total sample, the differences between the mean EQ-5D estimates using either calculation method (CLDQ-NAFLD domains or SF-6D) did not exceed 0.012 in all studied subgroups (by age, sex, obesity, T2D, and fibrosis stage) (Table 2).

**Figure:**
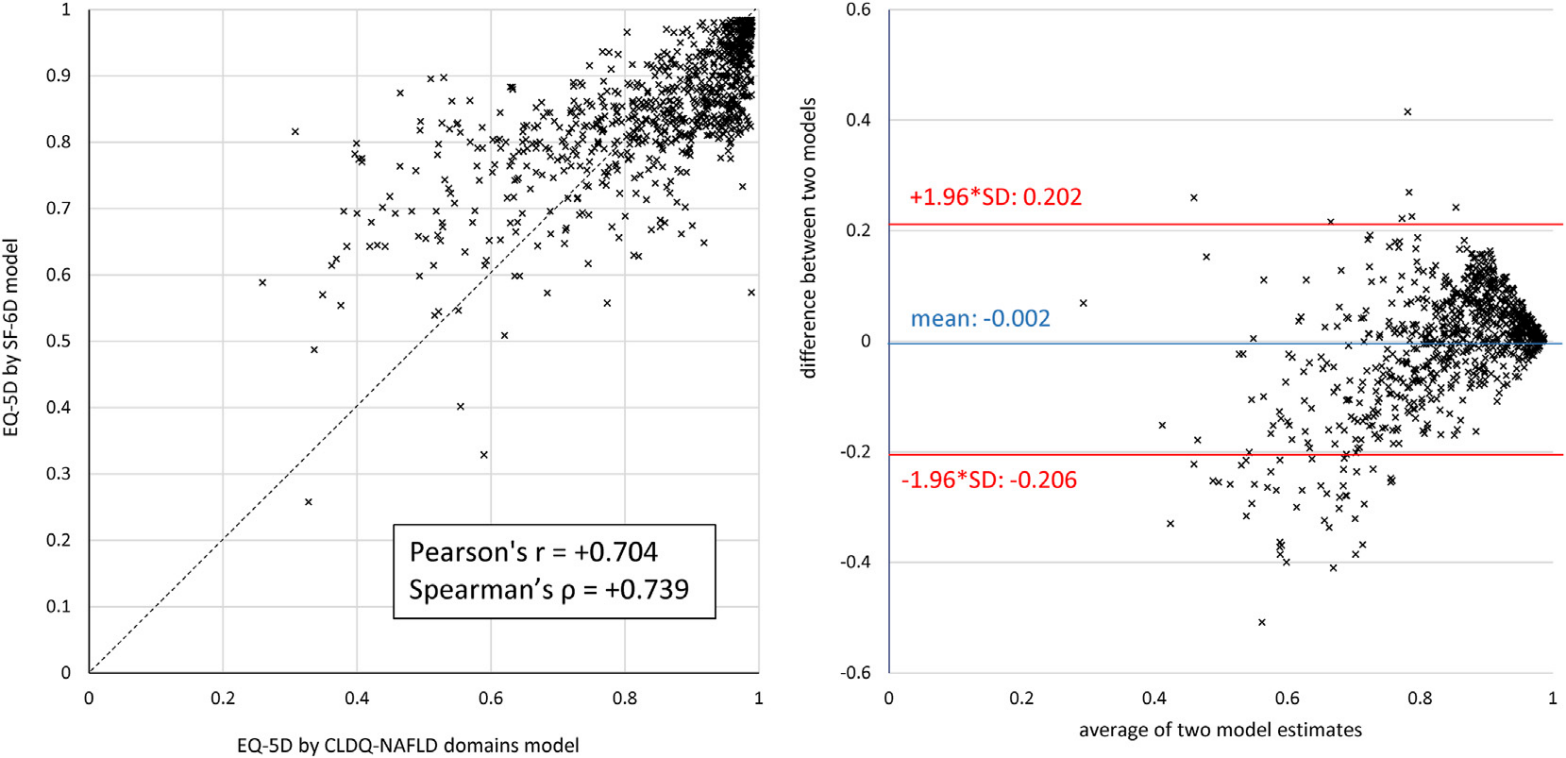
Concordance between 2 cross-walk algorithms for EQ-5D (CLDQ-NAFLD domain scores-based model and SF-6D-based model). Left panel: Bland-Altman plot; right panel: scatter plot with correlation coefficients.

**Table 2:** Modeled EQ-5D Scores in the Study Sample and its Subgroups

Subject group	EQ-5D mapping algorithm based on:	n	Mean EQ-5D estimate	SD of EQ-5D estimate
All noncirrhotic MASH	CLDQ-NAFLD total score	878	0.848	0.135
	CLDQ-NAFLD domain scores	878	0.851	0.146
	SF-6D score	883	0.853	0.097
<65 y	CLDQ-NAFLD total score	661	0.841	0.140
	CLDQ-NAFLD domain scores	661	0.844	0.155
	SF-6D score	664	0.852	0.101
≥65 y	CLDQ-NAFLD total score	217	0.868	0.114
	CLDQ-NAFLD domain scores	217	0.871	0.114
	SF-6D score	219	0.859	0.082
Female	CLDQ-NAFLD total score	489	0.820	0.140
	CLDQ-NAFLD domain scores	489	0.824	0.151
	SF-6D score	492	0.836	0.095
Male	CLDQ-NAFLD total score	389	0.882	0.119
	CLDQ-NAFLD domain scores	389	0.884	0.132
	SF-6D score	391	0.875	0.095
Non-Hispanic White	CLDQ-NAFLD total score	621	0.851	0.134
	CLDQ-NAFLD domain scores	621	0.852	0.147
	SF-6D score	625	0.853	0.095
Non-White race/ethnicity	CLDQ-NAFLD total score	247	0.839	0.136
	CLDQ-NAFLD domain scores	247	0.849	0.144
	SF-6D score	248	0.855	0.101
Nonobese (BMI <30)	CLDQ-NAFLD total score	172	0.868	0.128
	CLDQ-NAFLD domain scores	172	0.876	0.136
	SF-6D score	175	0.867	0.104
Obese (BMI ≥30)	CLDQ-NAFLD total score	706	0.843	0.136
	CLDQ-NAFLD domain scores	706	0.845	0.148
	SF-6D score	708	0.850	0.094
No type 2 diabetes	CLDQ-NAFLD total score	289	0.855	0.133
	CLDQ-NAFLD domain scores	289	0.859	0.150
	SF-6D score	288	0.863	0.095
Type 2 diabetes	CLDQ-NAFLD total score	589	0.844	0.135
	CLDQ-NAFLD domain scores	589	0.847	0.144
	SF-6D score	595	0.849	0.097
F1B/F2	CLDQ-NAFLD total score	336	0.847	0.143
	CLDQ-NAFLD domain scores	336	0.849	0.158
	SF-6D score	337	0.853	0.105
F3	CLDQ-NAFLD total score	542	0.848	0.129
	CLDQ-NAFLD domain scores	542	0.852	0.138
	SF-6D score	546	0.853	0.091

Using the CLDQ-NAFLD domain scores-based model, the EQ-5D scores in those subgroups were as follows (mean [SD]): 0.871 (0.114) in ≥65 years, 0.844 (0.154) in <65 years, 0.884 (0.132) in male, 0.824 (0.151) in female, 0.852 (0.147) in non-Hispanic White, 0.849 (0.144) in all other ethnic groups combined, 0.845 (0.148) in obese, 0.876 (0.136) in nonobese, 0.847 (0.144) in T2D, 0.859 (0.150) in non-T2D, 0.852 (0.138) in F3, and 0.849 (0.158) in F1B or F2 (Table 2).

In multivariate regression analyses, independent association of estimated EQ-5D scores with demographic and clinical parameters was similar between the CLDQ-NAFLD-based and the SF-6D-based models and included sex (lower scores in female patients), BMI (lower scores in patients with higher BMI), and history of psychiatric disorders (as collected from patients’ medical history) (*P* < .05) (Table 3). In addition, both EQ-5D estimates were found to be significantly negatively correlated with alkaline phosphatase (Spearman’s ρ = −0.135 and −0.143, respectively), liver stiffness by transient elastography (ρ = −0.094 and −0.090, respectively), and enhanced liver fibrosis scores (ρ = −0.083 and −0.091, respectively) (*P* < .05) (Table A2).

**Table 3:** Independent Association of Estimated EQ-5D Scores With Demographic and Clinical Parameters (Linear Regression Model)

Predictor of estimated EQ-5D score	EQ-5D estimate by the CLDQ-NAFLD-based model		EQ-5D estimate by the SF-6D-based model	
	Beta ± SE, ×100	*P*	Beta ± SE, ×100	*P*
Age, per y	0.068 ± 0.046	.14	0.012 ± 0.030	.69
Male (ref: female)	5.39 ± 0.96	<.0001	3.36 ± 0.62	<.0001
Non-Hispanic white (ref: non-white race)	−0.07 ± 1.07	.95	−0.25 ± 0.69	.72
BMI, per unit	−0.206 ± 0.072	.0044	−0.172 ± 0.047	.0003
Type 2 diabetes	−0.68 ± 1.03	.51	−0.68 ± 0.67	.31
Fibrosis stage F3 (ref: F1B/F2)	0.08 ± 0.98	.94	0.03 ± 0.64	.96
History of psychiatric disorders	−6.63 ± 0.96	<.0001	−5.25 ± 0.63	<.0001
History of clinically overt fatigue or asthenic conditions	−1.65 ± 1.90	.39	1.66 ± 1.24	.18

In the models, for presentation purposes, all EQ-5D score values were transformed to range of 0–100 (from original 0–1).

## Discussion

The types of PRO instruments utilized in clinical trials frequently vary from those used to assess HRQL to those used in cost-utility and other kinds of economic analyses. For the latter, instruments to assess health utilities or patients’ preferences are commonly used. In this context, generic preference-based instruments like the EQ-5D are popular but are not always employed in clinical trials due to questionnaire burden and regulatory bodies mandating the use of disease-specific instruments. If these scores are not available, mapping algorithms could offer a way to accurately approximate health utility scores even when a preference-based measure is not included in a particular study. In the current study, we reported an assessment of EQ-5D scores in patients with MASH using 2 different mapping methodologies based on widely used and extensively validated nonpreference-based PRO instruments.

There were several findings in this study. First, calculating EQ-5D from CLDQ-NAFLD and SF-36 (SF-6D) provided almost identical group scores, with the average difference not exceeding 0.01. Furthermore, there was no evidence of bias of either algorithm in any of the studied subgroups, although both algorithms seemed to perform better (as the difference between the estimates was smaller) at higher scores closer to perfect health. This provides further validation of the recently published study that reported calculating EQ-5D using CLDQ-NAFLD domain scores.^27^

In addition, our study provided some details about health utilities in baseline data of patients with MASH enrolled in a phase 3 clinical trial. We found that, regardless of the methodology used, patients of female gender as well as those with obesity or T2D had lower health utility scores, which is expected and consistent with the existing knowledge. On the other hand, estimated EQ-5D scores were not different between F1B/F2 and F3 patients with MASH included in this study. In the subsequent multivariate analysis that was used to control for potential confounders, only female sex, higher BMI, and having psychiatric comorbidities were independently associated with lower EQ-5D scores, again, regardless of the algorithm applied. The absolute EQ-5D estimates for the F3 group (0.852 and 0.853 by the 2 algorithms) were slightly higher than those reported for patients with F3 in one study (0.835 in F3 vs 0.819 in F4)^28^ but were expectedly significantly lower than those reported for individuals with excellent health (based on self-assessment of health on a 1–5 scale [excellent, very good, good, fair, poor]) (0.951, including 0.942 for men and 0.961 for women).^30^

Accurate health utility estimates are critical for economic evaluations when integrated into cost-effectiveness models. These models help policymakers allocate resources efficiently by comparing the costs of health-care interventions with their benefits, such as quality-adjusted life years or DALYs, determining the true value of interventions, and allow for better-informed decisions on which treatments or programs offer the greatest benefit relative to their cost. In the context of MASH, cost-effectiveness models may evaluate the value of different screening strategies, the cost vs effectiveness of interventions the choice of which is no longer limited to lifestyle, with available pharmacotherapy options including targeted treatment of steatohepatitis (eg, resmetirom) and other scenarios of early interventions in the context of varying progression rates of this heterogeneous disease.

The weakness of this study is the lack of the entire spectrum of patients with metabolic dysfunction-associated steatotic liver disease, including those without fibrosis or those with cirrhosis; the latter is particularly important given that the most profound impairment of HRQL is typically observed in this patient population. Additionally, the data used in this analysis come from patients enrolled in a phase 3 clinical trial after meeting the study’s inclusion/exclusion criteria and may not be representative of MASH patients seen in real-world gastroenterology and hepatology practices or those cared for by primary care providers. In particular, patients with various comorbidities were excluded, which limited our ability to account for their impact on health utilities. Further studies are needed to cover those gaps in the understanding of the disease burden.

In summary, both cross-walk algorithms for calculation of the EQ-5D utility scores in MASH patients were estimable with CLDQ-NAFLD or SF-36 instruments. A high positive correlation was seen between the total score and subgroup estimates using either method; this suggests confidence in their use to calculate quality-adjusted outcomes for cost-effectiveness models when direct health utility measures are not available.
