# Supplementary material for: Assessments of Health Utilities in Patients With Metabolic Dysfunction-Associated Steatohepatitis: Cross-Walk Between Disease-Specific Chronic Liver Disease Questionnaire, Short Form SF-6D, and EuroQol EQ-5D Instruments
Source: Gastro Hep Adv. 2025 Feb 7;4(6):100642. doi: 10.1016/j.gastha.2025.100642 (PMC12277723; doi:10.1016/j.gastha.2025.100642)
Supplement: Supplementary Table 1 and 2 [file mmc1.docx]

**Supplementary Table 1.** Demographic and clinical parameters of the historic study used for training the SF-6D-based model [28].

|  | Male | Female | *P* | All |
| --- | --- | --- | --- | --- |
| N | 649 | 974 |  | 1623 |
| Age, years | 56.5 ± 9.2 | 58.8 ± 8.5 | <.0001 | 57.8 ± 8.8 |
| Age ≥65 years | 138 (21.3%) | 285 (29.3%) | .0003 | 423 (26.1%) |
| Non-Hispanic White | 386 (59.5%) | 594 (61.0%) | .54 | 980 (60.4%) |
| Non-Hispanic Black | 9 (1.4%) | 11 (1.1%) | .65 | 20 (1.2%) |
| Hispanic | 76 (11.7%) | 160 (16.4%) | .0083 | 236 (14.5%) |
| Asian | 173 (26.7%) | 198 (20.3%) | .0029 | 371 (22.9%) |
| Other race/ethnicity | 4 (0.6%) | 8 (0.8%) | .64 | 12 (0.7%) |
| Enrolled in the U.S. | 322 (49.6%) | 584 (60.0%) | <.0001 | 906 (55.8%) |
| BMI, kg/m2 | 33.5 ± 6.1 | 33.4 ± 6.9 | .60 | 33.5 ± 6.6 |
| Obese (BMI >30) | 460 (70.9%) | 639 (65.6%) | .0261 | 1099 (67.7%) |
| Type 2 diabetes | 467 (72.0%) | 731 (75.1%) | .16 | 1198 (73.8%) |
| Hypertension | 442 (68.1%) | 666 (68.4%) | .91 | 1108 (68.3%) |
| Hyperlipidemia | 402 (61.9%) | 606 (62.2%) | .91 | 1008 (62.1%) |
| Fibrosis stage F3 | 333 (51.3%) | 439 (45.1%) | .0137 | 772 (47.6%) |
| Fibrosis stage F4 | 316 (48.7%) | 535 (54.9%) | .0137 | 851 (52.4%) |
| HbA1C, % | 6.56 ± 1.17 | 6.66 ± 1.20 | .17 | 6.62 ± 1.19 |
| Total cholesterol, mg/dL | 167.5 ± 40.0 | 182.2 ± 40.3 | <.0001 | 176.3 ± 40.8 |
| HDL, mg/dL | 43.7 ± 12.7 | 50.4 ± 14.0 | <.0001 | 47.7 ± 13.9 |
| LDL, mg/dL | 89.7 ± 34.2 | 99.2 ± 34.3 | <.0001 | 95.5 ± 34.5 |
| Triglycerides, mg/dL | 177.5 ± 112.4 | 166.0 ± 94.0 | .0355 | 170.5 ± 101.8 |
| ALT, U/L | 63.4 ± 37.3 | 52.2 ± 34.8 | <.0001 | 56.7 ± 36.2 |
| AST, U/L | 52.5 ± 27.4 | 52.9 ± 32.2 | .40 | 52.8 ± 30.3 |
| ALP, U/L | 85.9 ± 33.3 | 98.1 ± 35.5 | <.0001 | 93.2 ± 35.1 |
| CAP, dB/m | 326.4 ± 57.2 | 316.6 ± 52.9 | .0008 | 320.5 ± 54.8 |
| FIB-4 score | 2.52 ± 1.67 | 2.56 ± 1.59 | .32 | 2.54 ± 1.62 |
| ELF score | 10.3 ± 1.0 | 10.4 ± 1.0 | .0261 | 10.4 ± 1.0 |
| Liver stiffness by TE, kPa | 20.6 ± 13.4 | 19.1 ± 12.3 | .0266 | 19.7 ± 12.7 |
| SF-6D utility | 0.710 ± 0.140 | 0.662 ± 0.133 | <.0001 | 0.681 ± 0.138 |
| EQ-5D utility | 0.853 ± 0.134 | 0.810 ± 0.147 | <.0001 | 0.827 ± 0.144 |

**Supplementary Table 2.** Correlations (Spearman’s) of EQ-5D estimates with clinical and laboratory parameters.

| Parameter | EQ-5D estimate by the CLDQ-NAFLD-based model | | EQ-5D estimate by the SF-6D-based model | |
| --- | --- | --- | --- | --- |
|  | rho | *P* | rho | *P* |
| Age | 0.013 | .69 | 0.001 | .97 |
| BMI (kg/m2) | -0.155 | <.0001 | -0.156 | <.0001 |
| HbA1c (%) | -0.081 | .0170 | -0.055 | .10 |
| Total cholesterol (mg/dL) | -0.004 | .91 | -0.007 | .85 |
| HDL-C (mg/dL) | -0.037 | .27 | -0.058 | .08 |
| LDL-C (mg/dL) | 0.026 | .45 | 0.018 | .59 |
| Triglycerides (mg/dL) | -0.048 | .16 | -0.019 | .57 |
| ALT (U/L) | 0.041 | .22 | 0.071 | .0347 |
| AST (U/L) | 0.008 | .80 | 0.023 | .50 |
| **ALP (U/L)** | **-0.135** | **<.0001** | **-0.143** | **<.0001** |
| Fibroscan CAP (dB/m) | 0.012 | .73 | -0.013 | .71 |
| MRI-PDFF (%) | -0.025 | .50 | -0.017 | .64 |
| **Liver stiffness by TE (Fibroscan, kPa)** | **-0.094** | **.0059** | **-0.090** | **.0084** |
| **ELF score** | **-0.083** | **.0145** | **-0.091** | **.0070** |
| FIB-4 score | 0.031 | .35 | 0.036 | .28 |
